# Dietary Habits, Diet Quality, Nutrition Knowledge, and Associations with Physical Activity in Polish Prisoners: A Pilot Study

**DOI:** 10.3390/ijerph19031422

**Published:** 2022-01-27

**Authors:** Aureliusz Kosendiak, Piotr Stanikowski, Dorota Domagała, Waldemar Gustaw, Monika Bronkowska

**Affiliations:** 1 Department of Physical Education and Sport, Wroclaw Medical University, 51-601 Wroclaw, Poland; aureliusz.kosendiak@umw.edu.pl; 2 Department of Plant Food Technology and Gastronomy, Faculty of Food Science and Biotechnology, University of Life Sciences in Lublin, 20-704 Lublin, Poland; waldemar.gustaw@up.lublin.pl; 3 Department of Applied Mathematics and Computer Science, Faculty of Production Engineering, University of Life Sciences in Lublin, 20-612 Lublin, Poland; dorota.domagala@up.lublin.pl; 4 Institute of Health Sciences—Collegium Salutis Humanae, University of Opole, 45-060 Opole, Poland; monika.bronkowska@uni.opole.pl

**Keywords:** physical activity, prisoners, dietary habits, food frequency consumption, nutrition knowledge

## Abstract

Physical activity and healthy diets are determinants of the health of convicts who are deprived of freedom. However, little research has focused on these two aspects concurrently. The aim of this study is to analyze the relationships between the level of prisoners’ physical activity and their dietary habits, diet quality, and nutritional knowledge. The cross-sectional study was conducted with 226 prisoners. The inclusion criteria included male sex, age 18–65 years, and consent for the research. We excluded the subjects who met any of the following conditions: female sex, unwillingness to participate, and dangerous prisoner status. The KomPAN questionnaire was used to assess the prisoners’ eating habits, diet quality, and level of nutrition knowledge. The “Last 7d” short form of the International Physical Activity Questionnaire (IPAQ) was used to assess the prisoners’ level of physical activity. A significant correlation between the prisoners’ age and their levels of physical activity was found (*p* = 0.008). Prisoners who were aged 18–29 and over 40 declared a low level of physical activity, whereas those aged 30–40 reported a high or medium level. The level of physical activity of the surveyed prisoners was not significantly correlated with their dietary habits, the quality of their diet, or their nutrition knowledge.

## 1. Introduction

The penalty of deprivation of liberty is aimed at preventing the inmate from committing another offense. The procedure that is employed in order to achieve this goal is based on the stimulation of the convict’s willingness to cooperate in the rehabilitation process, sense of responsibility, and need to comply with applicable laws. In Poland, the penalty of deprivation of liberty is carried out in prisons or detention centers under the authority of the Minister of Justice [1].

The serving of a sentence of imprisonment has an impact on the mental and physical health of prisoners. The low physical activity resulting from the constant confinement, the limited access to physical work, and an improper diet are important factors leading to the deterioration of the prisoners’ health [2,3]. Psychiatric and digestive pathologies are the health problems that affect inmates most commonly [4]. An increased risk of diet-related diseases, e.g., overweight [5,6], obesity [5,6], diabetes [7,8,9], atherosclerosis [8,10], and arterial hypertension [4,8,10], is observed as well. The development of this type of disorder is promoted by many other factors that are not directly related to physical inactivity or inadequate nutrition, e.g., primarily drug use, alcohol abuse, and smoking [11]. Probably, the meals that are served in prisons, the possibility of buying additional food in canteens, and the limited possibilities of physical activity have negative health effects as well. Consequently, from 42 to 75% of men increase their body weight during imprisonment [5]. The study results indicate that a higher average weight gain is characteristic of female prisoners than male prisoners [6]. These factors influence the prisoner’s implementation of adequate nutrition principles or regular physical activity in the prison’s confinement conditions.

It is worth emphasizing, however, that legal regulations indicate that prisoners in Poland should not reject a meal that has been provided by the prison administration. Nevertheless, prisoners are free to make their own choices about the consumption of foods in two cases. First, they are allowed to purchase additional food in prison canteens at least 3 times per month. Secondly, they can receive food packages from their relatives once per month, which can contain up to 6 kg of food products and up to 9 L of beverages [1]. Unfortunately, there are no data on the scale of the food purchases that are made by Polish prisoners. Data from one of the prisons in Australia show that up to 30.5% of the energy that is supplied from the prisoners’ food may come from products that are purchased by the prisoners themselves. Australian inmates could purchase additional snack foods weekly (‘buy-ups’) [12]. However, research results indicate that the quality of the nutrition in Polish prisons needs to be improved. This is associated with the low catering budget [13], imprecise legal provisions regulating the issue of nutrition [1], and the need to train prison staff in appropriate diet planning [13]. A study that was conducted in a group of 307 male participants in an addiction treatment program in an American prison reported that only 6.7% of the inmates exhibited healthy diet choices [14]. Hence, educational programs are being organized, increasingly frequently, in order to educate adult prisoners [15] and juvenile offenders [16] in this regard. The information on nutrition was provided through lectures, discussion, vegetable garden designs, and wellness newsletters presenting nutritious recipes. After completion of the program, the participants declared improvement in their general health and dietary practices [15]. It is recognized internationally that the deprivation of freedom is an opportunity to introduce beneficial health options, not only in terms of nutrition, but also in the field of physical activity. Simultaneous nutritional and fitness programs have been reported to bring good health effects [17].

It can be concluded that the presently discussed situations that are related to the quality of the prisoner’s health and life are complementary. The health status and health needs of the individual are objective, whereas the expectations as to whether these needs can be satisfied by the prison are, unfortunately, subjective. Physical activity is undertaken by inmates on a voluntary basis and is performed during a designated time that is free from duty. The possibilities of taking up specific forms of physical activity depend on the sports and recreational facilities that are available in prisons and the offers from the sports clubs that are present in penitentiary institutions [18]. As reported by Mohan et al. [19], there is a strong relationship between the physical activity that is undertaken by prisoners and the quality of their lives, as physical exercise improves emotional functioning and helps to display emotions. The systematic activation of motor organs, adjusted to one’s fitness, enhances psychophysical abilities, minimizes aggressive behavior, and reduces the levels of stress and anxiety. It also exerts an impact on the prisoners’ social attitudes and value systems [20]. Studies that were conducted in French prisons have shown that physical activity is the main factor preventing the development of abdominal obesity in inmates [21]. As reported by Johnson et al. [22], high levels of physical activity (>60 min/day) are not sufficient to eliminate weight gain during incarceration. Nevertheless, inmates with high physical activity gain significantly less body weight compared to those who are not physically active.

Errors in the nutrition of inmates may result in the consolidation of bad eating habits and, in combination with a low level of physical activity, increase their risk of the development of lifestyle diseases. Serving a sentence of imprisonment is aimed at rehabilitation facilitating the return to life in society. Knowledge of the principles of healthy eating and the implementation of proper eating habits is one of the elements of this process.

The aim of this study is to analyze the relationships between the level of the prisoners’ physical activity and their dietary habits, diet quality, and nutrition knowledge.

## 2. Materials and Methods

### 2.1. Study Design and Participants

The present cross-sectional study was conducted at the beginning of 2020 in two penitentiary institutions that are located in western Poland. The first stage, the recruitment of respondents, lasted 1 month. Finally, 226 prisoners were qualified for the study. The inclusion criteria included male sex, age 18–65 years, and consent for the research. We excluded subjects who met any of the following conditions: female sex, unwillingness to participate, and dangerous prisoner status. Data were collected in the second stage, which lasted 1 month. The first author of this manuscript (A.K.) instructed the prison staff on the principles of the collection of the questionnaires. Additionally, the prisoners were instructed on the way of completion of each of the questionnaires. Two questionnaires in Polish were used: the International Physical Activity Questionnaire (IPAQ) and the Questionnaire of Dietary Habits and Nutrition Beliefs (KomPAN). The collected data were verified in the last stage, which lasted 5 months. Both of the questionnaires that were returned by the respondents were subjected to a preliminary analysis in order to check the completeness of the answers and the verification questions. Afterwards, the responses qualified 211 prisoners for the proper stage of the study. Within this group, 100 of the prisoners were detained in a closed ward for recurrent inmates in the Detention Center in Wrocław and 111 of the group were first-time prisoners in a closed ward in the Rawicz Prison. The survey was voluntary and anonymous. It was approved by the Bioethics Committee at the Medical University of Wrocław (approval no. KB-2720/2020).

### 2.2. Assessment of the Level of Physical Activity

The “Last 7d” short form of the International Physical Activity Questionnaire (IPAQ) was used to assess the level of physical activity. As shown by Polish research [23], the completion of the questionnaire by the respondents themselves may substantially overestimate the type of and time devoted to physical activity; hence, after previous training by our research team, prison workers were engaged in filling in the questionnaires. Energy expenditure was expressed in MET units, i.e., an equivalent of the resting metabolism that is equal to energy expenditure corresponding to the consumption of 3.5 mL of oxygen per kilogram of body weight per minute. The questionnaire assessed the intensity of the physical activity in agreement with the following MET values: 3.3 MET = low intensity, 4.0 MET = moderate intensity, and 8.0 MET = high intensity. The physical activity levels were also classified into three categories: low (LPAL), moderate (MPAL), and high (HPAL), according to the scoring system that is provided by the IPAQ [24].

### 2.3. Dietary Habits, Diet Quality and Nutrition Knowledge Level

The KomPAN questionnaire, which was validated for the Polish population ranging from adolescents to the elderly with varied health statuses [25], was used to assess the prisoners’ dietary habits, diet quality, and nutrition knowledge. The respondents who were confined in cells with other inmates filled in the questionnaire on their own. The questionnaire consisted of four parts comprising, in total, 111 questions that were grouped into the following topics: dietary habits, frequency of food consumption, nutrition beliefs, lifestyle, and personal data.

The nutrition knowledge was assessed based on the answers that were given by the respondents to 25 test questions with a varying degree of difficulty. The level of nutrition knowledge was determined based on the number of correct answers, as specified by the questionnaire’s analysis instructions: 0–8 = insufficient, 9–16 = sufficient, and 17–25 = good.

The quality of the diet was assessed on the basis of the answers to 24 questions about the frequency of food consumption. On their basis, two indices were calculated; i.e., the “Pro-Healthy Diet Index” and the “Non-Healthy Diet Index”. Next, based on the sum of the consumption frequency (times/day), the intensity of each index was assigned as follows:

- the “Pro-Healthy Diet Index”: pHDI-10 of 0–6.66—low; 6.67–13.33—moderate; 13.34–20—high;
- the “Non-Healthy Diet Index”: nHDI-14 of 0–9.33—low; 9.34–18.66—moderate; 18.67–28—high.

### 2.4. Statistical Analysis

The data were analyzed statistically with the use of the Microsoft Excel and Statistica 13.1 (Statsoft, Kraków, Poland) software packages. The values of the continuous parameters were presented as the mean value and standard deviation (SD) and the categorical ones were shown as the number and percentage. The missing observations have been replaced with the most common value. The age was divided into the following groups: 18 to 29, 30 to 39, 40 to 49, and 50 to 65. The Body Mass Index was split according to the guidelines of the WHO, but for the purpose of calculations the underweight and normal weight values were combined and all of the types of obesity constituted one group. The χ^2^ (chi-square) test of independence and the Kruskal–Wallis H test were used to analyze the relationship between the qualitative variables and the analysis of variance for the quantitative variable (the years of detention only; the analysis of height and weight was performed by analyzing the categorized BMI). The strength of the relationships between the level of physical activity and the analyzed variables was determined by the calculation of Cramer’s V coefficient. The analysis was performed at a significance level of 0.05 (the relationship between the variables was considered to be statistically significant when *p* < 0.05). The use of correspondence analysis facilitated the graphical presentation of the statistically significant correlations between the dependent variables. In addition, the results of the correspondence analysis were also interpreted in terms of a taxonomic analysis. For this purpose, on the basis of the calculated values of the coordinates of the points representing the analyzed categories, they were grouped using a hierarchical cluster analysis (HCA). Clustering was performed by means of the Ward distance matrix that formed on the basis of the Euclidean distance.

## 3. Results

Table 1 presents the characteristics of the survey respondents. The mean age of the prisoners was 37.10 ± 10.07 years and most of them declared a primary (31.75%) or secondary (61.61%) level of education. The vast majority of the respondents (70.62%) did not do professional sports before imprisonment.

### 3.1. Dietary Habits, Diet Quality and Nutrition Knowledge Level

The dietary habits of the prisoners are presented in Table 2. Most of the respondents (41.23%) declared the consumption of three meals per day. A large group (28.44%) comprised prisoners who consumed four meals per day. The frequency of eating between meals varied considerably. Mainly sweet-tasting foods, e.g., fruit, sweetened dairy beverages and desserts, and sweet snacks, were indicated by the respondents most frequently. As shown by the question about sweetening hot drinks, 35.55% of the respondents indicated that they did not use sugar or sugar substitutes. More than half of the prisoners (65.87%) participating in the study declared a varied frequency of adding salt to their dishes. More than 80% of the respondents did not follow any special diet.

The frequency of the consumption of the selected food products is presented in Table 3. As declared by the respondents, hot beverages such as coffee and tea were usually consumed by the prisoners several times per day. A low frequency of the consumption of sweetened carbonated/non-carbonated beverages was declared by the prisoners, as more than half of the respondents (53.08%) either did not consume such beverages at all or consumed them 1–3 times per month. The consumption of energy drinks was similarly low. Almost ¾ of the prisoners (72.98%) declared no consumption at all or a frequency of 1–3 times per month. The majority of the respondents (27.49%) indicated the consumption of fruit juices 1–3 times per month and 32.70% declared no consumption of vegetable juices. A much higher frequency was declared in the case of the consumption of sweets, as 38.86% of the respondents consumed sweet products several times per week.

The results of the questions on nutrition knowledge are presented in Table 4. The inmates most often assessed their nutrition knowledge as sufficient (44.08% of the responses) or good (35.55% of the responses). Based on the 25 test questions, it was shown that the majority of the respondents (63.98%) had a sufficient level of nutrition knowledge.

Table 5 presents the diet quality results. The Pro-Healthy Diet Index was low in the case of 89.10% of the respondents. Similarly, the Non-Healthy Diet Index was low in 95.26% of the participants of the study.

### 3.2. Associations of Dietary Habits, Food Consumption Frequency, and Nutrition Knowledge with the Level of Physical Activity

**Figure 1:**
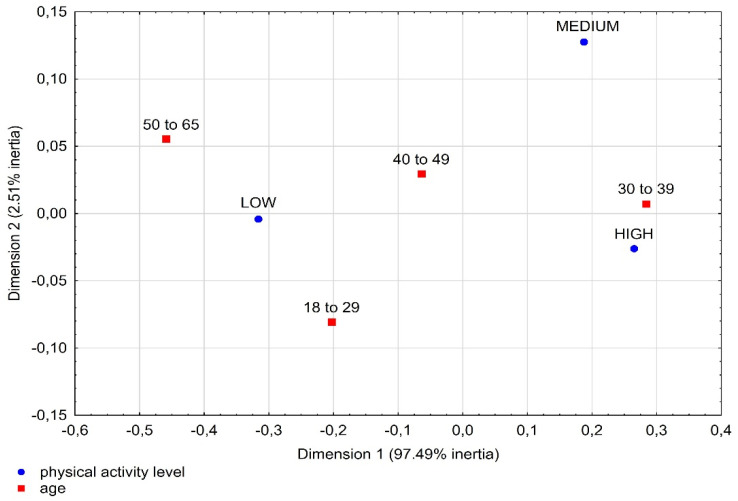
A biplot of the first two axes of correspondence analysis illustrating various physical activity levels and how they relate to specific age groups.

**Figure 2:**
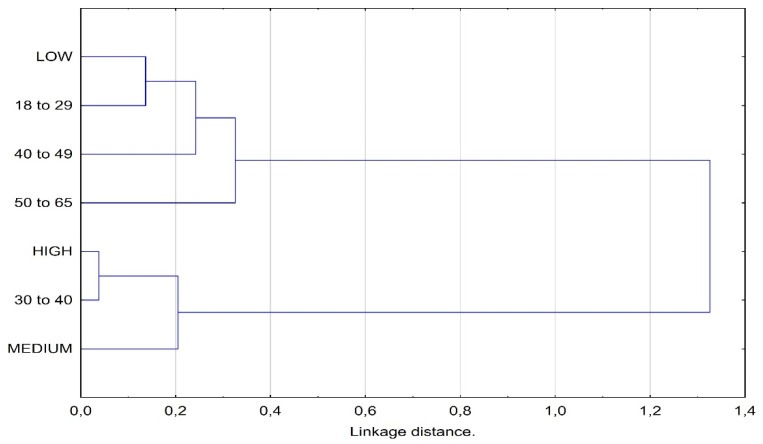
Dendrogram showing clustering of the analyzed categories.

The age of the respondents had a significant impact on their levels of physical activity (χ^2^ = 17.274, *p* = 0.008, Cramer’s coefficient V = 0.202). The graphical representation of the correlations of the level of physical activity with age was facilitated by the use of correspondence analysis (Figure 1). It is evident that the vertical axis separates the groups of prisoners with a low physical activity level from the prisoners with a high or medium level of physical activity. A low level of physical activity was characteristic for prisoners aged 18–29 and over 40. The respondents aged 30–40 represented a high or moderate level of physical activity. Figure 2 shows the results of grouping the analyzed categories by the means of the cluster analysis HCA.

No statistically significant relationships were found between the level of physical activity and the other variables.

## 4. Discussion

The results of the present study showed no effect of the level of physical activity on the level of nutrition knowledge, diet quality, or dietary habits that were declared by the prisoners. In turn, there was a correlation of physical activity with age.

The main motive for the inmates to undertake physical activity was the need to improve or maintain their physical fitness [3]. The structure of the level of physical activity that is shown in the present study (LPAL 44.80%, MPAL 10.43%, HPAL 45.50%) differed from the results of studies that were conducted in Italy [26], where 15.5%, 27.7%, and 56.8% of the prisoners were characterized by low, medium, and high physical activity, respectively, and results from other prisons in Poland [27] (45.4%, 35.3%, and 19.3%, respectively). Drug users entering prison had high levels of fitness and physical activity before admission, often gained from walking. Their walking activity reduced when they entered prison, posing a challenge to maintaining healthy activity levels [28]. As reported by Mannocci et al. [26], the level of physical activity increases with the age of the inmates and the length of their sentences. It is likely that the inmates with long-term sentences and older individuals feel the need to organize their interests and activities in order to improve the way in which they spend their time. The present observations showed a low level of physical activity in the group of prisoners who were aged 40–50 years and over 50. The low level of physical activity in the prisoners who were aged over 50 years may be mainly related to their poor physical and mental health, which is characteristic for this age group [29]. LPAL, which was characteristic of the respondents at the age of 18–29 years, may be associated with difficulties in adaptation in the prison environment, which are typical of young prisoners [30] and those who start serving their sentence [27]. The initial phase of imprisonment does not encourage the undertaking of regular physical activity and irregular forms of intense exercise help to release tension. A probable cause of the undertaking of more regular physical activity later during the imprisonment period is the gradual adaptation to the prison environment and an attempt to mitigate the effects of isolation [27].

Nearly 42% of the respondents were characterized by excessive body weight. This result is very similar to the data that are reported in the population of Canadian prisoners [31], but the obesity rates in the present study were substantially lower (18% vs. 45%). In turn, 38% of the prisoners had a BMI in a healthy range, which was lower than that which is found in the general Polish population (48%) [32]. Choudhry et al. [33] have shown that the BMI of prisoners in the UK increases over the first 6 imprisonment months and then declines until the 12th month. As reported by other authors, the average BMI of convicts who are serving a sentence in prisons in the USA is 28.7 and increases during the imprisonment time in 75% of prisoners [34]. The most common causes of body weight gain include intrinsic characteristics (age), extrinsic factors (diet and activity levels), and the prison regime (purposeful activity). High levels of stress and depressive symptoms may also contribute to weight gain [5,35]. Importantly, BMI should not be the only indicator of health risk that is associated with the composition and weight of the body in prisoners. For instance, a high BMI value that is associated with high muscle mass does not have comparable health implications to the case of the same BMI that is associated with high adipose tissue content [36]. The proportion of fat in the prisoners’ body compositions may be lower than that which is found in the general population due to the high popularity of bodybuilding/weightlifting activities inducing such changes [37,38].

The declaration of the consumption of three meals per day which was stated by the majority of the respondents (41.23%) is mainly related to the legal regulations specifying the number of meals that must be served in Polish prisons. Similar policies regarding the number of meals in prisons are implemented in other countries as well, e.g., the US [39,40], England [41], and the Balkan countries [42]. A higher number of meals (four) are served for juvenile detainees in the UK [43] and adult prisoners in Norway [44]. A large group in the present study comprised prisoners declaring the consumption of four (28.44%) or five meals per day (25.12%). This may imply that the respondents buy food in the canteen (at least three times per month) or get food packages from their relatives (once per month) [1]. Hence, the financial status of the convicts and their immediate family may have an indirect impact on the structure and frequency of their food consumption. In our unpublished studies that were conducted in 86 Polish prisons, we have shown a very wide range of food that the prisoners can buy in canteens [45]. Over 90% of such facilities were found to sell fruit, vegetables, unsweetened dairy products, sweetened dairy products, sweets, salty snacks, nuts, seeds, sugar, juices, and drinks. It was also possible to purchase energy drinks in 37% of the prisons. Given the results of research that was conducted in teenagers (*n* = 53,312) in Korea [46], which showed a link between the consumption of energy drinks and depressive moods and suicidal thoughts, limitation of the possibility to purchase such beverages in prison canteens should be considered. Similarly disturbing is the information about the 2-fold higher incidence of suicidal thoughts in American soldiers drinking one or more energy drinks per day, compared to those who do not consume such products [47]. This issue is important, as higher suicide rates have been determined in prisoners than in the general population [48]. Therefore, since almost half of the respondents (44%) declared the consumption of energy drinks, the potential negative effects of the consumption of such beverages by prisoners should be considered. In Poland, these beverages are especially popular with students of junior and senior high schools, as 67% of teenagers declare the consumption of energy drinks. Young people doing sports consume energy drinks more frequently than those with low physical activity (77% vs. 23%) [49]. In contrast, Gallucci et al. [50] showed no differences in the consumption of this group of products between athletes and non-athletes in American colleges. Our results have also indicated that energy drink consumption was similarly low among inmates with low and high levels of physical activity. Such drinks have a high price; therefore, the decision to buy them may be influenced not only by the level of physical activity but also by the prisoner’s financial status. In our research, we found a high frequency of the consumption of hot drinks. This may be related to the consumption of very strong black tea, i.e., the so-called “*czaj*”, which is used in Polish prisons as a stimulant. The greatest popularity of this drink was noted before the 1990s, after which it was gradually replaced by drugs [51]. The high frequency of the consumption of hot drinks that was reported by the prisoners in the present study has also been reported by other authors. As shown by Elger [52], prisoners in Switzerland drink 1.5 ± 2 cups of coffee per day and 1.7 ± 2 cups of tea. In Balkan prisons, coffee is more popular than tea [42]. It is often used as a currency, which may provoke jealousy and conflicts between inmates.

As reported by various authors, the level of nutrition knowledge in the group of athletes is higher or similar to that of physically inactive subjects [53] and increases through physical activity studies [54]. In the present study, we did not find significant correlations between the level of nutrition knowledge and the level of physical activity. Sufficient nutrition knowledge was exhibited by 63.98% of all respondents. A comparison of this result with the findings of a study that was conducted by Bieniek-Walenda et al. [55] using the same methodology showed no statistically significant differences. The authors showed that 74% of acute coronary syndrome patients had a “sufficient” level of nutrition knowledge. They also reported a higher level in patients with higher education. Polish pharmacists exhibit a significantly higher level of nutrition knowledge, with 70% characterized by a “good” level [56]. Learning about healthy dietary habits is important to prisoners. As reported by MacDonald et al. [30], over 90% of the young convicts in European prisons declare a need to improve their knowledge about healthy diets. Some results suggest that prisoners are aware of the necessity of pro-health consumer choices, but the meals that are served in prisons do not provide them with such an opportunity [57]. Therefore, it is extremely important that the Central Board of Prison Service, prison staff, and scientists take steps to improve prisoners’ access to healthy food.

The present results regarding the diet quality, based on the pHDI-10 values, differ from findings that have been reported by other authors who analyzed this issue in Polish professional athletes aged 36–65 years [58]. The Pro-Healthy Diet Index was low in 65% of the respondents. In the present study, almost 91% of prisoners in the HPAL group and 89% of all of the prisoners exhibited a low value of the Pro-Healthy Diet Index. In the analysis of these results, it should be borne in mind that prison meals are prepared in accordance with planned menus and prisoners can consciously choose only food purchased in the prison and food packages from their relatives. This may have a crucial impact on the quality of their diet.

### Limitations

This study presents certain limitations that must be explained. First, the study was conducted only on the prison population of one country, so it provides detailed information only about that particular population and should be reproduced in other countries. It should be emphasized that the present analyses were conducted in only two prisons. Taking into account the differences in the sports infrastructure and the number of organized sports activities between prisons, the level of the prisoners’ physical activity should be checked in a larger number of penitentiary institutions. Moreover, the study only looked at men. In subsequent studies, it is worth conducting similar studies among people of both sexes. Additionally, comparisons of the results that were obtained by gender would probably be very valuable.

It should be emphasized that the survey results were obtained from respondents who were deprived of liberty and most of them had a low level of education. Therefore, the present conclusions should not be referred to the general population.

## 5. Conclusions

The results of the present study showed no effect of the level of physical activity on the level of nutrition knowledge, diet quality, or dietary habits that were declared by the prisoners. In turn, there was a correlation of physical activity with the age of the respondents and the frequency of their consumption of energy drinks.

In our opinion, there is a need to conduct further research in order to assess the effects of education in the field of physical activity and nutrition. This knowledge could help prisoners to understand the importance and benefits of physical activity and proper nutrition for their health. The findings on the prisoners’ dietary habits and food consumption frequency may provide guidelines for prison staff to improve the quality of prison meals and help to introduce changes in the range of the food products that are available in prison canteens. Obviously, having access to healthy food does not guarantee that prisoners will only make pro-health consumer choices.

## Figures and Tables

**Table 1:** Lifestyle and personal data of prisoners by physical activity level.

	LPAL (*n* = 93)	MPAL (*n* = 22)	HPAL (*n* = 96)	Total (*n* = 211)	*p*-Value
Age (No. and %)					0.008
18–29	25 (11.85%)	3 (1.42%)	18 (8.53%)	46 (21.80%)	
30–39	29 (13.74%)	12 (5.69%)	54 (25.59%)	95 (45.02%)	
40–49	17 (8.06%)	4 (1.90%)	16 (7.58%)	37 (17.54%)	
50–65	22 (10.43%)	3 (1.42%)	8 (3.79%)	33 (15.64%)	
Age (Mean ± SD)	38.37 ± 11.99	38.00 ± 9.63	35.65 ± 7.76	37.10 ± 10.07	N.A.
Years of detention (Mean ± SD)	5.97 ± 6.87	6.11 ± 7.94	4.66 ± 5.83	5.38 ± 6.53	0.208
Education (No. and %)					0.096
Primary	34 (16.11%)	3 (1.42%)	30 (14.22%)	67 (31.75%)	
Lower secondary	25 (11.85%)	8 (3.79%)	23 (10.90%)	56 (26.54%)	
Upper secondary	25 (11.85%)	11 (5.21%)	38 (18.01%)	74 (35.07%)	
Higher	9 (4.27%)	0 (0%)	5 (2.37%)	14 (6.64%)	
Height (Mean ± SD)	177.84 ± 8.37	180.38 ± 4.64	178.49 ± 7.23	178.00 ± 7.54	N.A.
Weight (Mean ± SD)	85.15 ± 14.77	88.18 ± 11.58	84.61 ± 13.80	85.22 ± 14.00	N.A.
Body mass index (Mean ± SD)	26.96 ± 4.47	27.14 ± 3.62	26.56 ± 3.97	27.00 ± 4.15	N.A.
Body mass index (No. and %)					0.804
<18.5 (underweight)	0 (0%)	0 (0%)	2 (0.95%)	2 (0.95%)	
18.5–24.9 (normal weight)	41 (19.43%)	7 (3.32%)	34 (16.11%)	82 (38.86%)	
25.0–29.9 (overweight)	35 (16.59%)	10 (4.74%)	43 (20.38%)	88 (41.71%)	
>29.9 (obese)	17 (8.06%)	5 (2.37%)	17 (8.06%)	39 (18.48%)	
Smoking (No. and %)					0.240
Yes	53 (25.12%)	9 (4.27%)	46 (21.80%)	108 (51.18%)	
No	40 (18.96%)	13 (6.16%)	50 (23.70%)	103 (48.82%)	
Professional activity pre-reclusion (No. and %)					0.473
Yes	28 (13.27%)	4 (1.90%)	30 (14.22%)	62 (29.38%)	
No	65 (30.81%)	18 (8.53%)	66 (31.28%)	149 (70.62%)	

N.A. not analyzed, *p*-value obtained using the chi-square test to verify the relationship between categorized variables and the physical activity level or the analysis of variance for the years of detention.

**Table 2:** Dietary habits of prisoners by physical activity level.

	LPAL (*n* = 93)	MPAL (*n* = 22)	HPAL (*n* = 96)	Total (*n* = 211)	*p*-Value
How many meals do you usually consume daily?					0.769
1	2 (0.95%)	0 (0.00%)	1 (0.47%)	3 (1.42%)	
2	4 (1.90%)	0 (0.00%)	4 (1.90%)	8 (3.79%)	
3	42 (19.90%)	8 (3.79%)	37 (17.53%)	87 (41.23%)	
4	24 (11.37%)	8 (3.79%)	28 (13.27%)	60 (28.44%)	
5 or more	21 (9.95%)	6 (2.84%)	26 (12.32%)	53 (25.12%)	
Do you consume meals at regular times?					0.416
No	23 (10.90%)	2 (0,95%)	17 (8.06%)	42 (19,91%)	
Yes, but only some of them	34 (16.11%)	10 (4.74%)	43 (20.38%)	87 (41.23%)	
Yes, all of them	36 (17.06%)	10 (4.74%)	36 (17.06%)	82 (38.86%)	
How often do you snack between the meals?					0.345
Never	4 (1.90%)	3 (1.42%)	11 (5.21%)	18 (8.53%)	
1–3 times per month	24 (11.37%)	7 (3.32%)	16 (7.58%)	47 (22.27%)	
Once per week	10 (4.74%)	1 (0.47%)	14 (6.64%)	25 (11.85%)	
Few times per week	17 (8.06%)	5 (2.37%)	19 (9.00%)	41 (19.43%)	
Once per day	14 (6.64%)	3 (1.42%)	19 (9.00%)	36 (17.06%)	
Few times per day	24 (11.37%)	3 (1.42%)	17 (8.06%)	44 (20.85%)	
What types of food do you usually consume between the meals during the weekdays?					
Fruit	43 (22.28%)	13(6.74%)	38 (19.69%)	94 (48.70%)	0.372
Vegetables	18 (9.33%)	9 (4.66%)	21 (10.88%)	48 (24.87%)	0.215
Unsweetened dairy beverages and desserts	26 (13.47%)	4 (2.07%)	18 (9.33%)	48 (24.87%)	0.250
Sweetened dairy beverages and desserts	28 (14.51%)	5 (2.59%)	27 (13.99%)	60 (31.09%)	0.815
Sweet snacks	36 (18.65%)	9 (4.66%)	42 (21.76%)	87 (45.08%)	0.697
Savory snacks	22 (11.40%)	5 (2.59%)	23 (11.92%)	50 (25.91%)	0.983
Nuts, almonds, seeds	25 (12.95%)	5 (2.59%)	19 (9.84%)	49 (25.39%)	0.464
Other	10 (5.18%)	0 (0.00%)	7 (3.63%)	17 (8.81%)	0.193
Do you add any sugar to your hot beverages?					0.578
No	33 (15.63%)	6 (2.84%)	36 (17.06%)	75 (35.55%)	
Yes, I add one teaspoon of sugar (or honey)	22 (10.43%)	5 (2.37%)	19 (9.00%)	46 (21.80%)	
Yes, I add two or more teaspoons of sugar (or honey)	24 (11.37%)	8 (3.79%)	25 (11.85%)	57 (27.01%)	
Yes, I use sweeteners (low-caloric substitute for sugar)	16 (7.58%)	3 (1.42%)	14 (6.63%)	33 (15.64%)	
Do you add salt to your meals and sandwiches once prepared?					0.193
No	25 (11.85%)	6 (2.84%)	41 (19.43%)	72 (34.12%)	
Yes, but only sometimes	52 (24.64%)	12 (5.69%)	41 (19.43%)	105 (49.76%)	
Yes, I add salt to most of my meals	16 (7.58%)	4 (1.90%)	14 (6.64%)	34 (16.11%)	
Are you currently following a diet?					0.300
No	76 (36.02%)	17 (8.06%)	78 (36.97%)	171 (81.04%)	
Yes, as advised by my doctor for medical reasons	7 (3.32%)	1 (0.47%)	12 (5.69%)	20 (9.48%)	
Yes, it was my personal decision	10 (4.74%)	4 (1.90%)	6 (2.84%)	20 (9.48%)	

Each *p*-value was obtained by using the chi-square test to verify the relationship between each studied variable and the physical activity level.

**Table 3:** Consumption frequency of selected food by physical activity level.

	LPAL (*n* = 93)	MPAL (*n* = 22)	HPAL (*n* = 96)	Total (*n* = 211)	*p*-Value
Sweetened hot beverages (black tea, coffee, herbal or fruit teas)					0.978
Never	11 (5.21%)	4 (1.90%)	13 (6.16%)	28 (13.27%)	
1–3 times per month	8 (3.79%)	3 (1.42%)	6 (2.84%)	17 (8.06%)	
Once per week	7 (3.32%)	2 (0.95%)	6 (2.84%)	15 (7.11%)	
Few times per week	16 (7.58%)	3 (1.42%)	15 (7.11%)	34 (16.11%)	
Once per day	12 (5.69%)	3 (1.42%)	16 (7.58%)	31 (14.69%)	
Few times per day	39 (18.48%)	7 (3.32%)	40 (18.96%)	86 (40.76%)	
Sweetened carbonated or still beverages					0.091
Never	29 (13.74%)	6 (2.84%)	20 (9.48%)	55 (26.07%)	
1–3 times per month	22 (10.43%)	5 (2.37%)	30 (14.22%)	57 (27.01%)	
Once per week	14 (6.64%)	4 (1.90%)	17 (8.06%)	35 (16.59%)	
Few times per week	18 (8.53%)	2 (0.95%)	20 (9.48%)	40 (18.96%)	
Once per day	6 (2.84%)	0 (0.00%)	5 (2.37%)	11 (5.21%)	
Few times per day	4 (1.90%)	5 (2.37%)	4 (1.90%)	13 (6.16%)	
Energy drinks					0.175
Never	54 (25.59%)	9 (4.27%)	55 (26.07%)	118 (55.92%)	
1–3 times per month	15 (7.11%)	7 (3.32%)	14 (6.64%)	36 (17.06%)	
Once per week	7 (3.32%)	4 (1.90%)	8 (3.79%)	19 (9.00%)	
Few times per week	12 (5.69%)	0 (0.00%)	13 (6.16%)	25 (11.85%)	
Once per day	2 (0.95%)	0 (0.00%)	6 (2.84%)	8 (3.79%)	
Few times per day	3 (1.42%)	2 (0.95%)	0 (0.00%)	5 (2.37%)	
Fruit juices					0.622
Never	16 (7.58%)	3 (1.42%)	22 (10.43%)	41 (19.43%)	
1–3 times per month	22 (10.43%)	4 (1.90%)	32 (15.17%)	58 (27.49%)	
Once per week	23 (10.90%)	5 (2.37%)	14 (6.64%)	42 (19.91%)	
Few times per week	23 (10.90%)	6 (2.84%)	18 (8.53%)	47 (22.27%)	
Once per day	6 (2.84%)	2 (0.95%)	7 (3.32%)	15 (7.11%)	
Few times per day	3 (1.42%)	2 (0.95%)	3 (1.42%)	8 (3.79%)	
Vegetable juices or fruit and vegetable juices					0.402
Never	30 (14.22%)	5 (2.37%)	34 (16.11%)	69 (32.70%)	
1–3 times per month	28 (13.27%)	7 (3.32%)	24 (11.37%)	59 (27.96%)	
Once per week	14 (6.64%)	2 (0.95%)	16 (7.58%)	32 (15.17%)	
Few times per week	13 (6.16%)	2 (0.95%)	14 (6.64%)	29 (13.74%)	
Once per day	6 (2.84%)	3 (1.42%)	4 (1.90%)	13 (6.16%)	
Few times per day	2 (0.95%)	3 (1.42%)	4 (1.90%)	9 (4.27%)	
Sweets (confectionary, biscuits, cakes, chocolate bars, cereal bars, other)					0.901
Never	10 (4.74%)	2 (0.95%)	5 (2.37%)	17 (8.06%)	
1–3 times per month	17 (8.07%)	4 (1.90%)	19 (9.00%)	40 (18.96%)	
Once per week	16 (7.58%)	6 (2.84%)	13 (6.16%)	35 (16.59%)	
Few times per week	34 (16.11%)	7 (3.32%)	41 (19.43%)	82 (38.86%)	
Once per day	11 (5.21%)	2 (0.95%)	12 (5.69%)	25 (11.85%)	
Few times per day	5 (2.37%)	1 (0.47%)	6 (2.84%)	12 (5.69%)	

Each *p*-value was obtained using the chi-square test to verify the relationship between each studied variable and the physical activity level (for this purpose, the answers Once per day and Few times per day were combined where necessary).

**Table 4:** Nutrition knowledge of prisoners by physical activity level.

	LPAL (*n* = 93)	MPAL (*n* = 22)	HPAL (*n* = 96)	Total (*n* = 211)	*p*-Value
Self-reported nutrition knowledge level					0.500
Insufficient	17 (8.06%)	4 (1.90%)	17 (8.06%)	38 (18.01%)	
Sufficient	44 (20.85%)	7 (3.32%)	42 (19.91%)	93 (44.08%)	
Good	29 (13.74%)	11 (5.21%)	35 (18.59%)	75 (35.55%)	
Very good	3 (1.42%)	0 (0.00%)	2 (0.95%)	5 (2.37%)	
Nutrition knowledge level					0.367
Insufficient	25 (11.85%)	8 (3.79%)	25 (11.85%)	58 (27.49%)	
Sufficient	53 (25.12%)	13 (6.16%)	69 (32.70%)	135 (63.98%)	
Good	15 (7.11%)	1 (0.47%)	2 (0.95%)	18 (8.53%)	

Each *p*-value was obtained by using the chi-square test to verify the relationship between each studied variable and the physical activity level.

**Table 5:** Diet quality indexes by physical activity level.

	LPAL (*n* = 93)	MPAL (*n* = 22)	HPAL (*n* = 96)	Total (*n* = 211)	*p*-Value
Pro-Healthy Diet Index					0.362
Low	82 (38.86%)	19 (9.00%)	87 (41.23%)	188 (89.10%)	
Medium	11 (5.21%)	2 (0.95%)	8 (3.80%)	21 (9.95%)	
High	0 (0.00%)	1 (0.47%)	1 (0.47%)	2 (0.95%)	
Non-Healthy Diet Index					0.344
Low	90 (42.65%)	20 (9.48%)	91 (43.13%)	201 (95.26%)	
Medium	3 (1.42%)	1 (0.47%)	4 (1.90%)	8 (3.80%)	
High	0 (0.00%)	1 (0.47%)	1 (0.47%)	2 (0.95%)	

Each *p*-value was obtained by using the chi-square test to verify the relationship between each studied variable and the physical activity level.

## Data Availability

The research results presented are part of a large ongoing study which has not yet been completed. If you are interested in specific data, please contact the corresponding author.
